# Development and external validation of a nomogram prediction model based on quantitative coronary angiography for predicting ischemic lesions: a multi-centre study

**DOI:** 10.3389/fcvm.2025.1550550

**Published:** 2025-06-20

**Authors:** Shuai Yang, Shuang Leng, Zhouchi Wang, Jiang Ming Fam, Adrian Fatt Hoe Low, Ru-San Tan, Ping Chai, Lynette Teo, Chee Yang Chin, John C. Allen, Mark Yan-Yee Chan, Khung Keong Yeo, Aaron Sung Lung Wong, Soo Teik Lim, Qinghua Wu, Liang Zhong

**Affiliations:** ^1^ Department of Cardiology, Henan Provincial Chest Hospital, Zhengzhou, Henan, China; ^2^ National Heart Research Institute Singapore, National Heart Centre Singapore, Singapore, Singapore; ^3^ Cardiovascular Sciences Academic Clinical Programme, Duke-NUS Medical School, Singapore, Singapore; ^4^ Department of Cardiology, National University Heart Centre, Singapore, Singapore; ^5^ Yong Loo Lin School of Medicine, National University of Singapore, Singapore, Singapore; ^6^ Department of Diagnostic Imaging, National University Hospital, Singapore, Singapore; ^7^ Department of Cardiovascular Medicine, The Second Affiliated Hospital of Nanchang University, Nanchang, Jiangxi, China; ^8^ Department of Biomedical Engineering, National University of Singapore, Singapore, Singapore

**Keywords:** fractional flow reserve, ischemic lesions, quantitative coronary angiography, nomogram, prediction model

## Abstract

**Objectives:**

Quantitative coronary angiography (QCA) has significantly contributed to the diagnosis of coronary artery disease. This study aimed to construct and validate a QCA-based prediction model, represented as a nomogram, for predicting ischemic lesions defined by invasive fractional flow reserve (FFR) ≤ 0.80.

**Methods:**

In this multi-centre study, we enrolled 220 patients with 303 interrogated vessels who underwent FFR measurements during clinically indicated invasive coronary angiography. QCA predictors for ischemic lesions were extracted to construct a nomogram model using Least Absolute Shrinkage and Selection Operator (LASSO) regression analysis of the development set (*n* = 113 patients). An external validation (*n* = 107 patients) was performed to assess the nomogram model's discrimination and consistency.

**Results:**

Lesion length, minimal lumen diameter, stenosis flow reserve, percent diameter stenosis by visual estimation, and weight were included as predictors in the nomogram. The nomogram yielded an area under the curve (AUC) of 0.922 and 0.912 at per-vessel and per-patient levels, respectively, in the development set. In the validation set, it achieved an AUC of 0.915 and 0.912 at per-vessel and per-patient levels, respectively. Per-vessel accuracy, sensitivity, and specificity derived from the nomogram were 86.5%, 88.2%, 86.2% in the development cohort and 84.2%, 85.5%, and 83.1% in the validation cohort. For per-patient analysis, the corresponding values were 85.8%, 85.7%, 86.0% in the development cohort and 82.2%, 83.3%, 81.1% in the validation cohort.

**Conclusion:**

The nomogram may be useful for predicting ischemic lesions using QCA measurements and the LASSO regression algorithm, with external validation indicating potential predictive value in cardiology care settings.

## Graphical Abstract

**Figure:**
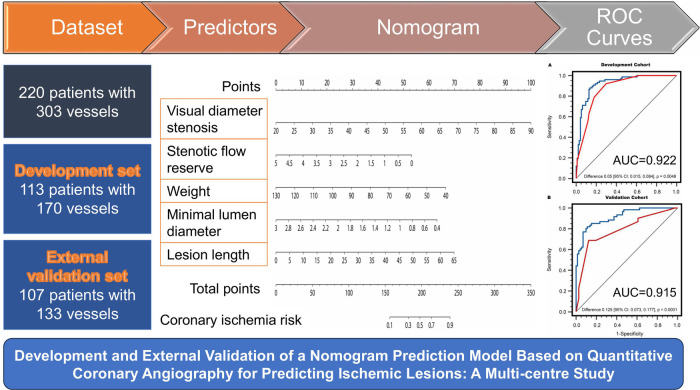
Development and external validation of a nomogram prediction model based on quantitative coronary angiography for predicting ischemic lesions: A multi-centre study.

## 1 Introduction

Atherosclerotic cardiovascular disease continues to be the leading cause of morbidity and mortality worldwide, despite significant improvements over the past few decades (1). The fractional flow reserve (FFR) measured during invasive coronary angiography (ICA) is currently considered the reference standard for the diagnosis of hemodynamically significant coronary stenosis (ischemic lesions) (2, 3). Prior studies had shown that the implementation of FFR-guided coronary intervention strategies can enable both a reduction in stent overuse and a significant improvement in long-term prognosis (3–7).

With the advancement of medical digital imaging and communication technology and new contour detection algorithms, quantitative coronary angiography (QCA), as an objective and high reproducibility computer-assisted technique, has made significant contributions to disease diagnosis and clinical decision-making by providing accurate measurements of each parameter and facilitating the effective evaluation of treatment efficacy (8–10). According to Academic Research Consortium-2 (11) and Japanese Association of Cardiovascular Intervention and Therapeutics (12) clinical expert consensus documents, the cardiovascular medical team was suggested to utilize QCA analysis to assess the anatomical structure of coronary stenosis and predict myocardial ischemia in conjunction with clinical data. In recent years, several hemodynamic parameters derived from QCA like stenotic flow reserve (SFR) and the ratio of lesion length to the fourth power of the minimal lumen diameter (LL/MLD^4^) have been confirmed to be associated with ischemic lesions (13, 14).

Accordingly, the aim of this study was to identify major risk predictors that correlate with ischemic lesions from QCA-derived parameters or demographic parameters. More importantly, we seek to construct and validate a diagnostic prediction model displayed as a novel nomogram to calculate individualized predictions of ischemic lesions risk defined by invasive FFR ≤ 0.80.

## 2 Methods

### 2.1 Study design and population

The study is a multi-centre trial designed to develop and validate a predictive model and evaluate the diagnostic accuracy of QCA-based model in identifying hemodynamically significant coronary artery disease (CAD) as defined by invasive FFR reference standard (**Graphical abstract**). This study received approval from the local institutional review boards at each participating centre. All prospectively enrolled patients provided individual informed consent, while consent was waived for the anonymized analysis of retrospective data. We prospectively enrolled 178 patients with suspected or known CAD at two tertiary centres in Singapore from September 2016 to October 2020 and retrospectively included 142 patients in China from January 2014 to December 2021. Patients who underwent ICA and FFR measurements and had at least one stenosis with percent diameter stenosis by visual estimation (DS_VE_) between 30% and 90% were included in this study for QCA analysis. Patients were not eligible if they had a history of coronary artery bypass grafting, myocardial infarction within 72 h of coronary angiography or severe heart failure (left ventricular ejection fraction <30%). Angiographic exclusion criteria were as follows: poor-quality coronary angiograms that precluded contour detection; interrogated vessels with in-stent restenosis; left main coronary artery disease; tight stenosis or tortuosity that prevented the FFR wire from crossing the lesion; severe vessel overlap or tortuosity at the stenotic segments; luminal reduction caused by myocardial bridge; and severe aortic stenosis and/or donor vessels supplying significant collaterals to chronic total occlusion vessels.

**Figure 1:**
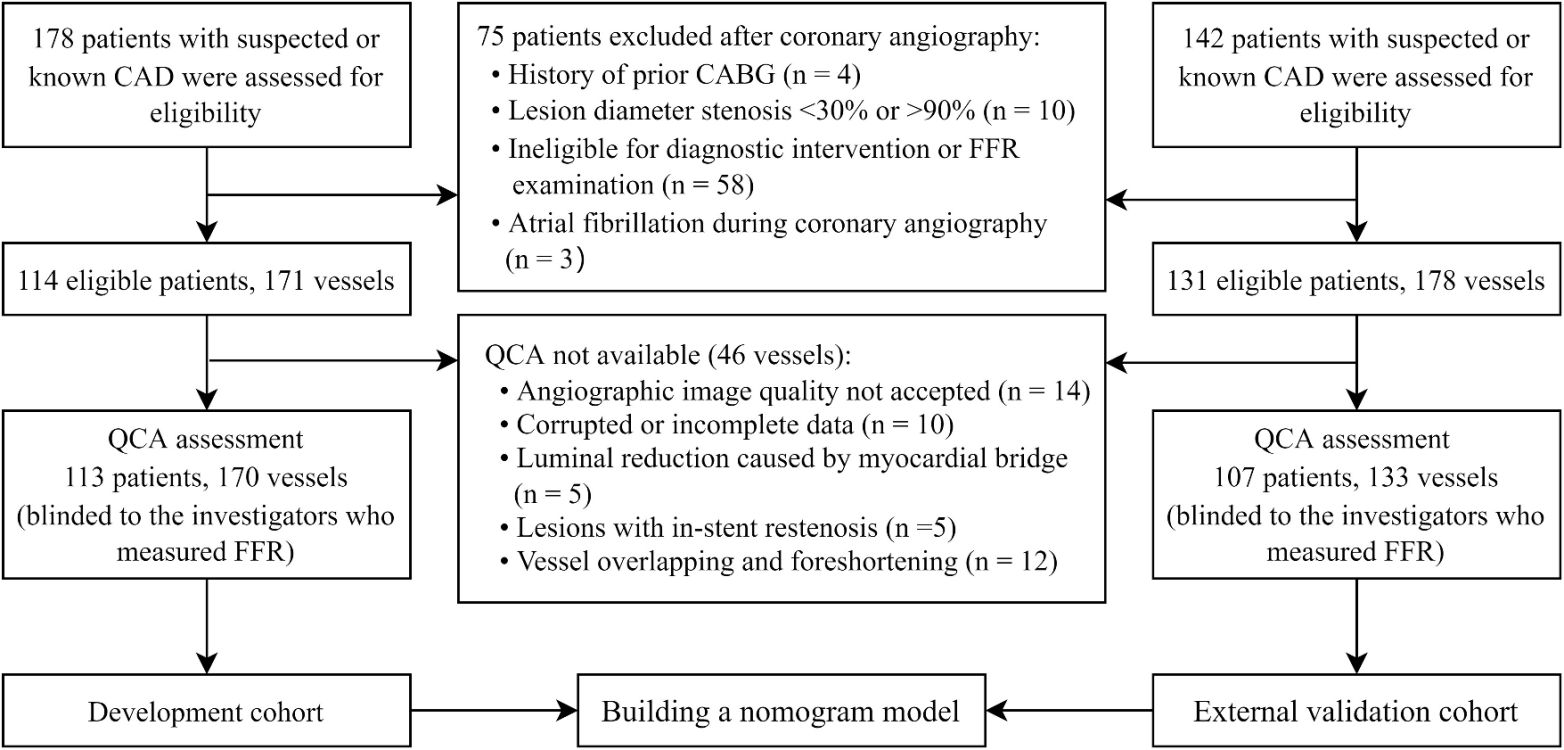
Flowchart of patient recruitment and study design.

Finally, the study included 220 patients with 303 interrogated vessels. Among the included participants, 113 patients with 170 vessels from Singapore were selected to develop the nomogram model, while the remaining 107 patients with 133 vessels from China were used to validate the model. The flowchart depicting the recruitment process and research design is shown in Figure 1.

### 2.2 ICA and FFR measurements

ICA and FFR measurements were performed according to standard guidelines (15) and the institutional protocol. FFR measurements, using the pressure wire provided by St. Jude Medical and ACIST Navvus, were conducted at least three times, with the lowest recorded value for each vessel being assessed (16). Each patient received adenosine intravenously (140–180 μg/kg/min) or through an intracoronary bolus (60–200 μg) to induce maximal hyperemia. A diagnosis of hemodynamically significant coronary stenosis was based on an invasive FFR value of ≤0.80.

### 2.3 Quantitative coronary angiography

The QCA analyses were conducted in a core catheterization laboratory using Qangio XA (version 7.3, Medis Medical Imaging System BV, Leiden, The Netherlands), with the analysts blinded to the ICA and FFR results. All QCA analyses were captured during the end-diastolic phase of the heart cycle, ensuring minimal movement of the target coronary vessel within the frame. For sequential lesions, the lesion with the highest degree of stenosis was selected to represent the vessel. A single frame was selected from the best available projection with the least foreshortening and overlap with other structures, demonstrating the most severe lumen narrowing. Calibration was performed using the catheter tip (16). Following the determination of a proximal start point and a distal end point of the coronary segment of interest, a vessel pathline was created, extending from the proximal to distal points. Subsequently, the vessel contour was automatically detected in accordance with the pathline. Both the pathline and vessel contour can be manually corrected as necessary.

### 2.4 Extraction of QCA features and demographic characteristics

**Figure 2:**
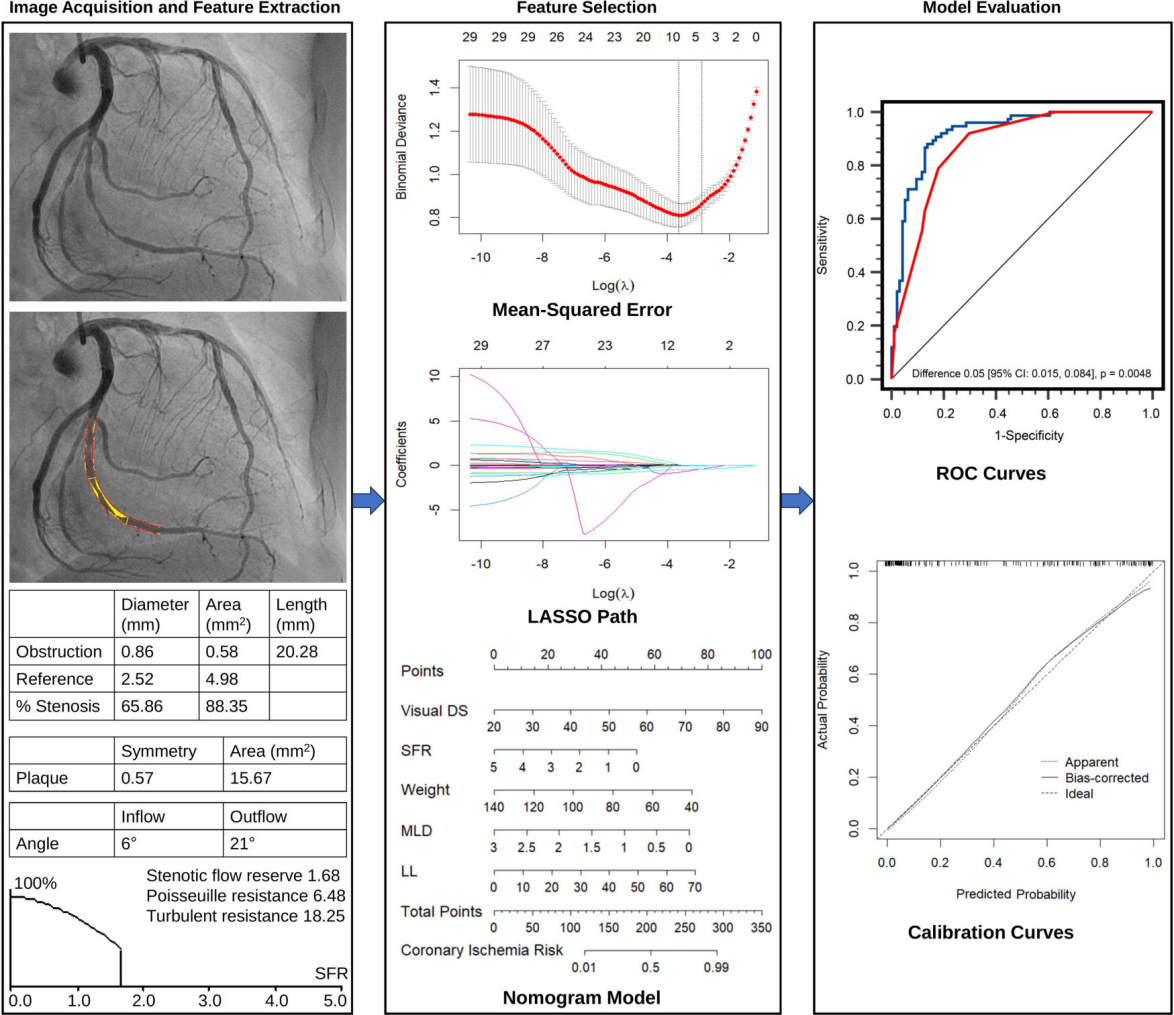
Overall flow chart of the study, including image acquisition and quantitative analysis, feature extraction, feature selection, nomogram model construction, and evaluation.

The QCA analyses produced a series of parameters, encompassing morphological, plaque characteristic, and hemodynamic aspects (Figure 2). The major morphological parameters obtained through QCA included: (1) **Lesion length (LL)**: the distance from the proximal lesion-free segment to the distal lesion-free segment; (2) **Reference diameter and area**: computer-generated estimates of the diameter and area at the site of lumen stenosis, assuming the absence of atherosclerotic lesions; (3) **Minimal lumen diameter (MLD)** and **minimal lumen area**: the minimum diameter and cross-sectional area measured at the site of maximal stenosis; (4) **Diameter stenosis (DS_QCA_, %)**: calculated as [1—(MLD/reference lumen diameter)] × 100%; and (5) **Area stenosis**: calculated as [1—(minimal lumen area/reference lumen area)] × 100%.

The extracted plaque characteristic parameters were as follows: (1) **Plaque area**: the plaque area within lesion length; (2) **Plaque symmetry**: the ratio between the plaque areas on both sides in a two-dimensional plane at the interrogated lesion, ranging from 0 (indicating complete asymmetry) to 1 (representing complete symmetry).

The following hemodynamic parameters extracted were obtained through QCA: (1) **LL/MLD^4^**: ratio of LL to the fourth power of MLD; (2) **Poiseuille resistance and turbulent resistance**: the laminar and turbulent resistance to blood flow through the stenosis, respectively; (3) **Stenotic flow reserve (SFR)**: the hemodynamic consequence of a stenosis (14) based on the assumption of a quadratic relationship between flow and pressure drop (across the stenotic region). Detailed lesion characteristics assessed by QCA and their definitions/descriptions are provided in Supplementary Table S1.

Clinical measurements and essential demographic characteristics, considered as potential risk predictors, were reviewed from electronic medical records. These included age, sex, height, weight, body mass index, blood pressure, symptoms of angina, and risk factors such as hypertension, diabetes, hyperlipidemia, current smoking, and family history of CAD. Additionally, the degree of stenosis by ICA (DS_VE_) was also considered an important morphological parameter.

In total, 29 parameters were extracted, including 13 demographic parameters (Table 1), 15 QCA features (Table 2 and Supplementary Material), and 1 ICA parameter (DS_VE_).

**Table 1:** Clinical characteristics of study subjects in the development cohort and validation cohort.

Variable	Development (*n* = 113)	Validation (*n* = 107)	*P* value
Age, years	60.0 ± 9.2	65.5 ± 8.5	<0.001
Female	31 (27.4)	32 (28.8)	0.816
Weight, kg	72.1 ± 16.4	65.0 ± 12.4	<0.001
Height, m	1.65 ± 0.09	1.64 ± 0.08	0.667
Body mass index, kg/m^2^	26.3 ± 4.8	23.9 ± 3.6	<0.001
Systolic blood pressure, mmHg	134.6 ± 18.0	134.4 ± 19.4	0.931
Diastolic blood pressure, mmHg	76.8 ± 10.2	76.7 ± 12.0	0.939
Hypertension	70 (61.9)	69 (64.5)	0.696
Hyperlipidemia	71 (62.8)	19 (17.8)	<0.001
Diabetes Mellitus	32 (28.3)	13 (12.1)	0.003
Current smoker	26 (23.0)	38 (35.5)	0.056
Symptoms with angina	60 (53.1)	58 (54.2)	0.869
Family history of CAD	12 (10.6)	20 (18.7)	0.090

Values are mea*n* ± SD or *n* (%). CAD, coronary artery disease.

**Table 2:** Characteristics of interrogated vessels in the development cohort and validation cohort.

Lesion characteristics	Development (*n* = 170)	Validation (*n* = 133)	*P* value
Anatomical parameters			
Reference diameter, mm	2.8 ± 0.6	2.9 ± 0.6	0.303
Reference area, mm^2^	6.5 ± 2.8	6.8 ± 3.0	0.347
Minimal lumen diameter, mm	1.3 ± 0.5	1.4 ± 0.5	0.115
Minimal lumen area, mm^2^	1.5 ± 1.3	1.7 ± 1.2	0.327
Lesion length, mm	20.0 ± 11.0	14.3 ± 6.4	<0.001
Inflow angle, deg	12.8 ± 7.4	17.3 ± 8.6	<0.001
Outflow angle, deg	12.0 ± 7.7	13.5 ± 7.4	0.078
Area stenosis, mm^2^	76.6 ± 14.1	76.1 ± 10.9	0.765
Plaque parameters			
Plaque symmetry	0.6 ± 0.3	0.6 ± 0.3	0.437
Plaque area	13.0 ± 8.0	9.9 ± 6.0	<0.001
Hemodynamic Parameters			
Turbulent resistance	16.4 ± 45.7	4.4 ± 10.1	0.001
Poiseuille resistance	5.9 ± 10.5	2.1 ± 2.7	<0.001
SFR	3.0 ± 1.3	3.3 ± 0.9	0.02
LL/MLD^4^, mm^−3^	47.7 ± 117.7	11.8 ± 19.6	<0.001
FFR	0.80 ± 0.14	0.81 ± 0.11	0.345
Vessels with FFR ≤0.80	76 (44.7)	61 (45.9)	0.841
Diameter stenosis of lesion, %			
DS by visual estimation (DS_VE_)	61.4 ± 18.3	71.6 ± 11.0	<0.001
DS by QCA (DS_QCA_)	54.0 ± 14.8	52.4 ± 11.0	0.284
DS_VE_ ≥ 70%	77 (45.3)	97 (72.9)	<0.001
DS_QCA_ ≥ 50%	95 (55.9)	78 (58.6)	0.629

Continuous variables are presented as mean ± SD or *n* (%). LL/MLD^4^, the ratio of lesion length to the fourth power of the minimal lumen diameter; DS, diameter stenosis; QCA, quantitative coronary angiography; FFR, fractional flow reserve; SFR, stenotic flow reserve.

### 2.5 Feature selection and prediction model building

The Least Absolute Shrinkage and Selection Operator (LASSO) regression was utilized to identify the most valuable features in the development set (Figure 2). The LASSO algorithm was implemented using the “glmnet” package in R software (version 4.2.1, R Project for Statistical Computing, Vienna, Austria). To determine the optimal penalty parameter (λ), a ten-fold cross-validated was performed. The value of λ corresponding to the most regularized and parsimonious model within one standard error of the minimum was selected (17). This λ was then applied in the LASSO model to compute the regression coefficients for each feature. Features with non-zero coefficients were selected for subsequent logistic regression analysis to assess their associations with functional coronary ischemia, following Harrell's guidelines. Collinearity of each selected variable by LASSO regression was diagnosed using the variance inflation factor, and variables with variance inflation factor >5 indicated significant multicollinearities (18). A nomogram visualizing the logistic regression model was constructed to predict the probability of functional coronary ischemia (Figure 2). This provides clinicians with an intuitive and quantitative method to identify suspected CAD patients with functionally significant stenosis.

According to coefficients in the nomogram model, the predicted probability of functionally significant coronary stenosis can be calculated using the following formula:

$$P(\mathrm{Functionally}\;\mathrm{Significant}\;\mathrm{Coronary}\;\mathrm{Stenosis})=\frac{1}{1+\exp(-(\beta_{0}+\beta_{1}x_{1}+\beta_{2}x_{2}+\cdots +\beta_{n}x_{n}))}$$

where $\beta_{0},\;\beta_{1},\;\beta_{2},\ldots ,\;\beta_{n}$ are the coefficients corresponding to the intercept and the respective variables in the final logistic regression model; $x_{1},x_{2},\ldots ,\;x_{n}$ are the observed values of those variables.

A flowchart illustrating the LASSO model training, validation, and performance evaluation process is provided in Supplementary Figure S1.

### 2.6 Development and validation sets

Datasets from two hospitals in Singapore constituted the development cohort, while datasets from one hospital in China were designated as an external validation cohort to assess the generalizability of the prediction model.

### 2.7 Reliability

For reliability assessment, a random sample of 50 patients with 50 coronary lesions was re-analyzed by the same observer at least one month after the initial assessment, and it was independently assessed by another observer. To evaluate both intra- and inter-observer reliabilities, intraclass correlation coefficients (ICCs) were calculated for each potential parameter. Following Landis and Koch's criteria, ICC values were categorized as follows: excellent agreement (ICC ≥ 0.75), good agreement (0.6 ≤ ICC < 0.75), moderate agreement (0.4 ≤ ICC < 0.6), and poor agreement (ICC < 0.40) (19).

### 2.8 Statistical analysis

Continuous variables were expressed as mean with standard deviation, and categorical variables were presented as counts and percentages. Student's *t*-test or one-way analysis-of-variance was employed for normally distributed variables, while the nonparametric Mann–Whitney *U* test was used for non-normally distributed variables. Binary variables were compared using the chi-squared test.

Receiver operating characteristic curve for the nomogram model was generated at the vessel level in both the development and validation sets. The probability of ischemic lesions for each patient was calculated based on the nomogram. Sensitivity, specificity, positive predictive value (PPV), negative predictive value (NPV), and accuracy were determined using the optimal threshold of the probability of ischemic lesions.

The Hosmer-Lemeshow test was used to assess the diagnostic consistency of the model and to create smooth-fitting curves that compare actual and predicted probabilities. A *P* value >0.05 indicated good agreement between the new model and standard diagnostic criteria. Calibration curves were plotted to demonstrate the consistency between predicted and actual probabilities of ischemic lesions (20). Decision curve analysis was utilized to determine the net benefit for patients at each threshold probability, assessing the utility and clinical value of predictive nomograms (21).

Two-sided *P* values < 0.05 were considered statistically significant. All analyses were conducted using IBM SPSS version 23.0 (SPSS Inc., Chicago, USA), MedCalc software version 20.100 (MedCalc Software Ltd, Ostend, Belgium), and R software version 4.2.1 (R Project for Statistical Computing, Vienna, Austria). The “rms” package of R software was employed to construct the nomogram and plot the calibration (Figure 2). Analyses were conducted both on a per-vessel basis and on a per-patient basis. For the per-patient analysis, the vessel with the most adverse clinical status (indicated by the minimum FFR and highest diameter stenosis) was chosen to represent each patient.

## 3 Results

### 3.1 Baseline patient and lesion characteristics

The clinical characteristics of the patients in the development cohort and external validation cohort are detailed in Table 1. The mean age of patients in the development cohort and the validation cohort was 60.0 ± 9.2 and 65.5 ± 8.3, respectively; 31 (27.4%) were women in the development cohort, and 32 (28.8%) in the validation cohort. The development group had a higher prevalence of hyperlipidemia and diabetes, higher body mass index, weight, and younger age compared to the validation group. The other baseline characteristics of the patients were similar between the development and validation groups.

Table 2 lists the characteristics associated with interrogated vessels in both the development and validation cohorts. The interrogated vessels in both cohorts exhibited similar MLD, DS_QCA_, SFR, and invasive FFR. FFR of ≤0.80 was measured in 76 vessels (45%) and 61 vessels (46%) in the development and validation cohorts, respectively.

### 3.2 Feature selection and model construction

**Figure 3:**
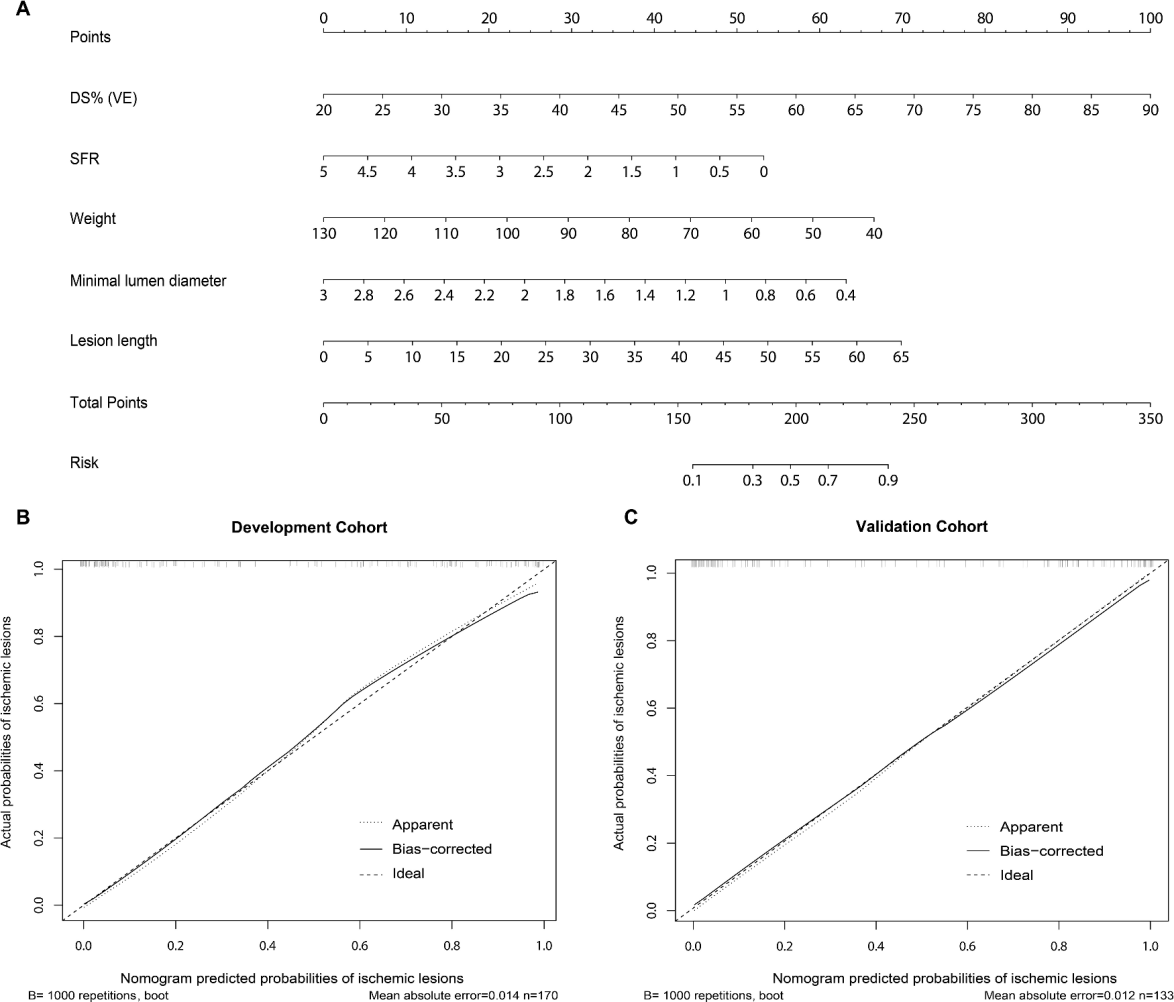
Nomogram and calibration performance of the model for predicting functionally significant stenosis. **(A)** Nomogram to estimate the risk of functionally significant stenosis in patients with suspected or known CAD. To use the nomogram, find the position of each variable on the corresponding axis, draw a line to the points axis for the number of points, add the points from all of the variables, and draw a line from the total points axis to determine the ischemic lesions probabilities at the lower line of the nomogram; The calibration curve of the nomogram model in estimating the risk of ischemic lesions in the **(B)** development cohort and **(C)** validation cohort. CAD, coronary artery disease; DS%, percent diameter stenosis; SFR, stenotic flow reserve; VE, visual estimation.

This study was designed based on the Transparent Reporting of a Multivariable Prediction Model for Individual Prognosis or Diagnosis (TRIPOD) guidelines (22). From the pool of 29 candidate features in the development cohort, the LASSO algorithm generated a total of 5 feature parameters with non-zero coefficients. These parameters included DS_VE_, weight, and 3 QCA-derived features (MLD, LL, SFR) (Figure 3 and Supplementary Table S2). Supplementary Figure S2 illustrates the generation of the optimal penalization coefficient lambda. Based on these results, we constructed a nomogram model to predict functionally significant coronary stenosis (Figure 3A).

### 3.3 Diagnostic performance of nomogram model for identifying significant stenosis

**Figure 4:**
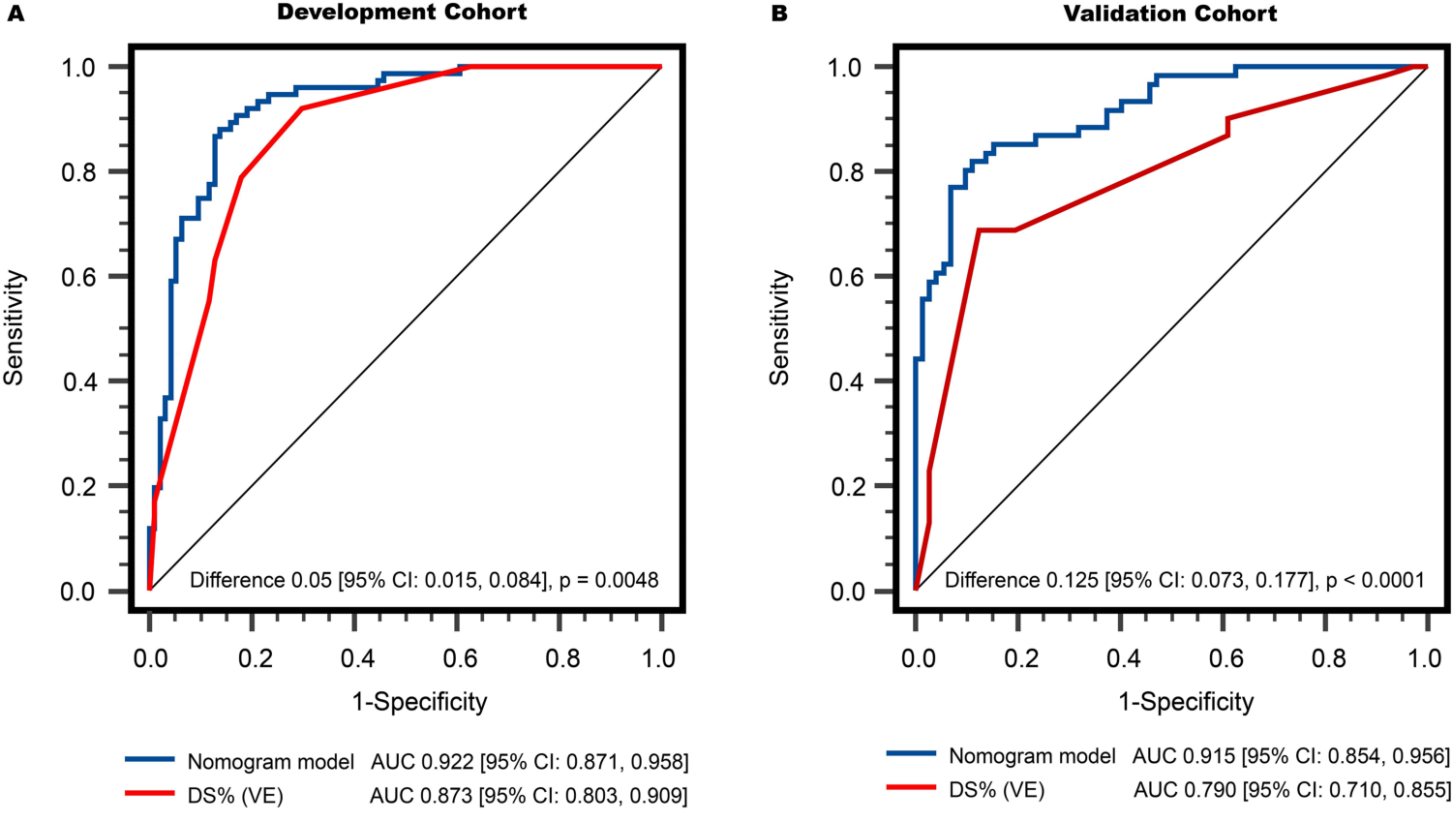
Comparison of vessel-level diagnostic performance in discriminating functionally significant stenosis: **(A)** development cohort, and **(B)** validation cohort. The AUCs of the nomogram model were both significantly higher than that of DS% (VE). AUC, areas under the receiver operator characteristics curve; DS%, percent diameter stenosis; QCA, quantitative coronary angiography; VE, visual estimation.

As shown in Figure 4A, in the development population, the vessel-level analysis demonstrated that the nomogram model exhibited discriminative ability to predict ischemic lesions with an area under the curve (AUC) of 0.922 (95% CI, 0.871–0.958). This performance was significantly higher than that of DS_VE_ (*P* = 0.005). In the validation population, the AUC for the nomogram model was also significantly larger than for DS_VE_ (0.915 vs. 0.790, *P* < 0.0001, Figure 4B). Similar results were observed in the patient-level analysis in both the development and external validation populations (Supplementary Figure S3).

The predicted probability can be calculated using following formula: Probability = 1/(1 + exp(−(−0.021 + 0.057 × LL (mm)—1.291 × MLD (mm) + 0.076 × DS_VE_ (%)−0.566 × SFR−0.039 × weight (kg)))). The risk probability of 0.5 was calculated as the cutoff value to identify physiologically significant coronary stenosis. In the development population, per-vessel diagnostic sensitivity and specificity were significantly larger with the nomogram model than that with DS_VE_ ≥ 70% (sensitivity: 88.2% vs. 78.9%; specificity: 86.2% vs. 81.9%). The diagnostic accuracy, PPV, and NPV of the nomogram model were 86.5%, 83.5%, and 89.0%, respectively, which were also significantly higher than DS_VE_ ≥ 70% (Table 3). In the validation population, the diagnostic sensitivity of the nomogram model was slightly lower than that of DS_VE_ ≥ 70% (sensitivity: 85.5% vs. 87.1%). However, specificity, accuracy, PPV, and NPV of the nomogram model significantly outperformed DS_VE_ ≥ 70% (83.1%, 84.2%, 81.5%, and 86.7% vs. 38.0%, 60.9%, 55.1% and 77.1%, respectively).

**Table 3:** Per-vessel diagnostic accuracy of nomogram model, and DS_VE_ in the development and validation sets.

Cohort	Metric	Nomogram model risk score ≥0.5	DS_VE_ ≥70%
Development cohort	Accuracy	86.5 (80.5–90.9)	80.6 (74.0–85.9)
	Sensitivity	88.2 (78.2–94.1)	78.9 (67.8–87.1)
	Specificity	86.2 (77.2–92.1)	81.9 (72.3–88.8)
	PPV	83.5 (73.1–90.6)	77.9 (66.8–86.3)
	NPV	89.0 (80.3–94.3)	82.8 (73.3–89.6)
Validation cohort	Accuracy	84.2 (77.0–89.5)	60.9 (52.4–68.8)
	Sensitivity	85.5 (73.7–92.7)	87.1 (75.6–93.9)
	Specificity	83.1 (71.9–90.6)	38.0 (27.0–50.4)
	PPV	81.5 (69.6–89.7)	55.1 (44.7–65.1)
	NPV	86.7 (75.9–93.4)	77.1 (59.4–89.0)

Metrics are expressed as percentage (95% confidence interval).

DS_VE_, diameter stenosis by visual estimation; PPV, positive predictive value; NPV, negative predictive value.

Additionally, in the development population, the nomogram model exhibited per-patient diagnostic sensitivity and specificity of 85.7% (95% CI, 76.0–92.9%) and 86.0% (95% CI, 72.6–93.7%), respectively, which were significantly larger than those of DS_VE_ ≥ 70%. Moreover, the nomogram model demonstrated higher accuracy, PPV, and NPV compared to DS_VE_ ≥ 70%. Patient-level analysis revealed similar diagnostic accuracy in the validation population (Supplementary Table S3).

To assess the model's performance across different coronary territories, we conducted a subgroup analysis comparing lesions in the left anterior descending (LAD) artery vs. non-LAD vessels. In the development cohort, the model achieved an AUC of 0.955 (95% CI, 0.892–0.986) for LAD lesions and 0.901 (95% CI, 0.808–0.959) for non-LAD lesions. Similarly, in the validation cohort, the AUC was 0.935 (95% CI, 0.863–0.976) for LAD and 0.919 (95% CI, 0.794–0.980) for non-LAD vessels. These results indicate that the model maintained strong and comparable discriminatory performance across both LAD and non-LAD territories in both cohorts (Supplementary Figure S4).

For vessel-level analysis, the calibration curves demonstrated good consistency between the predicted and actual probabilities of functionally significant stenosis for the nomogram model in both the development and validation cohorts (Figures 3B,C). Similarly, at the patient level, good calibration curves for risk estimation were observed in both the development and validation cohorts (Supplementary Figure S5). Meanwhile, the Hosmer-Lemeshow test indicated that the nomogram was well-fitted, showing no significant differences (all *P* > 0.05).

### 3.4 Decision curve analysis

Decision curve analysis was employed to assess the utility of different predictive models by calculating the clinical net benefit at various probability thresholds. In Supplementary Figure S6, the decision curve analysis curve demonstrates that the predictive nomogram (the green line), derived from the development set, exhibits higher net benefits compared to DS_VE_ when the probability threshold of ischemia ranges from 10%–80% in both the development and validation sets. This holds true for both the treat-all-patients and treat-none schedules. These findings indicate that the nomogram model is a reliable clinical tool for predicting functionally significant coronary stenosis.

### 3.5 Reliability

The ICC values for QCA parameter analysis indicated that 14 parameters exhibited excellent correlation, 4 demonstrated good correlation, 5 showed moderate correlation, and only 1 exhibited poor correlation for intra-observer reliability. Similarly, for inter-observer reliability, 7 parameters demonstrated excellent correlation, 9 exhibited good correlation, 5 demonstrated moderate correlation, and only 2 showed poor correlation. More importantly, the ICC values for the three QCA parameters in the predictive model—MLD, LL, and SFR—showed excellent intra- and inter-observer reliability with correlation coefficients of 0.94, 0.82, and 0.92, and 0.90, 0.76, and 0.84, respectively (Supplementary Table S4).

## 4 Discussion

In this multi-centre study, we observed that a nomogram model, constructed using QCA-derived parameter features, exhibited superior performance in identifying hemodynamically significant coronary stenosis defined by invasive FFR ≤ 0.80. In both the development cohort and the external validation cohort, the diagnostic accuracy at both the vessel and patient levels was significantly higher than that achieved with traditional DS_VE_ based on visual assessment, yielding notable insights into the potential clinical utility of the nomogram.

Angiographic physician visual assessment, being the most common method, has been routinely employed to determine stenosis severity and guide revascularization interventions (16). According to the 2021 ACC Guideline for Coronary Artery Revascularization, visually estimated DS severity of ≥70% for non-left main disease has been used to identify significant stenosis and guide revascularization strategy (23). An angiographically intermediate coronary stenosis is defined as a DS severity ranging from 40%–69%, often requiring additional investigation to determine physiological significance. However, it can be challenging to ascertain on the coronary angiogram, through simple visual assessment, which lesions cause ischemia. Reports indicate high frequencies of visual-functional mismatch between angiography and invasive FFR, signifying inaccuracy in assessing the functional significance of coronary stenosis, not only in the 50% to 70% category but also in the 70% to 90% angiographic severity category (24, 25). Similarly, prior studies (26–28) have demonstrated substantial discrepancies between DS_QCA_ ≥ 50% and physiological significance confirmed by invasive FFR ≤ 0.8. Therefore, it is reasonable to hypothesize that incorporating lesion geometry and physiological blood flow is more accurate in diagnosing ischemic lesions than relying solely on anatomical stenosis. The results of this study did indeed support our supposition.

To the best of our knowledge, this study is the first to apply the LASSO regression algorithm to select features and construct a nomogram model based on QCA for identifying ischemic lesions. The LASSO regression parameter selection algorithm is a crucial component of machine learning under artificial intelligence. Unlike other statistical modeling methods, the LASSO procedure enables compression estimation through a shrinkage property, leading to more stable variable selection and improved prediction accuracy and model interpretation (29–31). In this study, 29 candidate QCA-derived and demographic features were reduced to 5 potential predictors by evaluating the predictor-outcome association and applying the LASSO method to shrink the regression coefficients. This approach is more effective than selecting predictors solely based on their univariable association with the outcome (29, 30).

Among the QCA-based parameters, LL, MLD, and SFR were selected using LASSO. A study by Yong et al. (31) revealed a moderate correlation between 2-dimensional QCA parameters, such as MLD and minimal lumen area, with invasive FFR. SFR, denoting coronary flow reserve at a specific aortic pressure in the presence of stenosis, is calculated as the maximal to resting flow ratio based on Poiseuille's law of fluid dynamics. This parameter has demonstrated close associations with ischemia, as assessed through pharmacological stress echocardiography and perfusion imaging (32, 33). In the evaluation by Potter et al. (14), SFR exhibited modest predictive value for functionally significant coronary stenosis, as determined by invasive FFR. Nevertheless, the accuracy of these QCA features remains uncertain when externally validated in patients from different centres. In this study, we not only constructed a nomogram model incorporating several QCA-derived features to identify physiologically significant coronary stenosis but also externally validated the feasibility and accuracy of this nomogram, yielding excellent results. Notably, there was no significant difference in the diagnostic performance of the nomogram model in discriminating hemodynamically significant lesions in the development and validation cohorts (AUC 0.922 vs. 0.915, *P* > 0.05), underscoring its robustness and generalizability. It is noteworthy that the external validation cohort was derived from a real-world dataset obtained from a large-scale hospital. This is a crucial step in ensuring that the model's performance holds in diverse patient populations and clinical settings, reinforcing its applicability beyond the development cohort.

The construction of the nomogram represents a practical and user-friendly application of the study's findings. By converting complex regression coefficients into a visual tool, clinicians can readily estimate individualized probabilities of functionally significant coronary stenosis. Additionally, the nomogram's reliability was assessed through intra- and inter-observer reliability analyses, demonstrating excellent correlation for key QCA parameters (MLD, LL, and SFR). This underscores the reproducibility and consistency of the nomogram model in different clinical settings. The nomogram's potential impact on clinical decision-making is underscored by its superior performance in decision curve analysis. The consistently higher net benefits across various probability thresholds indicate that the nomogram could provide valuable guidance in determining the need for further diagnostic and therapeutic interventions.

The developed model serves as a valuable adjunct to traditional assessment methods, including FFR, rather than a complete replacement. Its primary utility lies in providing a comprehensive risk stratification tool that integrates multiple imaging and clinical parameters to identify patients at higher risk for significant coronary artery disease. In routine catheterization laboratory practice, the model can assist clinicians in several key ways. First, it supports clinical decision-making by evaluating lesion characteristics and predicting ischemic potential, thereby helping to prioritize which lesions warrant FFR assessment and which may be managed conservatively. Second, by identifying low-risk cases, the model has the potential to reduce unnecessary FFR testing, enhancing workflow efficiency and conserving resources. Lastly, it facilitates more personalized treatment planning by enabling tailored strategies based on individual risk profiles, which may contribute to improved patient outcomes through more targeted interventions.

This study has limitations. First, vessels with in-stent restenosis or side branches of bifurcation lesions with Medina type 1,1,1 were not assessed. Further research is needed to assess the generalizability of this nomogram model to the side branches of coronary bifurcation lesions. Second, this analysis was based on data from three institutions, which were primarily high-volume ICA centres. Additional centres with larger sample sizes are warranted to further validate the results. Third, the difference in QCA availability between the development and validation cohorts is primarily due to the nature of data collection. The development cohort was part of a prospective study, where QCA acquisition was pre-specified and systematically performed, resulting in near-complete data (only one case excluded). In contrast, the validation cohort was retrospective, and QCA data were not consistently available due to factors such as suboptimal image quality, incomplete data, presence of myocardial bridges, in-stent restenosis, and vessel overlapping and foreshortening. As a result, approximately 21% of patients in the validation cohort were excluded from QCA-based analyses. Fourth, in this study, the decision to proceed with FFR was made at the discretion of the interventionalist, based on the severity of stenosis, lesion characteristics, and clinical judgment. Patients with clearly non-significant or clearly severe stenoses—where FFR was deemed clinically unnecessary—were not subjected to FFR, resulting in a larger number of patients without FFR measurements, particularly in the development cohort. Fifth, future research avenues may explore the incorporation of additional QCA-derived parameters or the refinement of the model based on evolving technology and clinical insights. Long-term prospective studies could further elucidate the nomogram's impact on patient outcomes and its potential role in optimizing treatment strategies. Finally, this study did not include a comparison with the instantaneous wave-free ratio, a widely used non-hyperemic pressure index for assessing CAD.

## 5 Conclusions

Our study introduces a novel nomogram model based on QCA-derived parameters for predicting FFR-defined coronary ischemia. The model, validated externally, demonstrates superior diagnostic performance compared to traditional visual diameter assessment. By combining lesion geometry and physiological blood flow parameters, the nomogram provides a nuanced and personalized approach to predicting FFR-defined coronary ischemia.

## Data Availability

The original contributions presented in the study are included in the article/Supplementary Material, further inquiries can be directed to the corresponding authors.
