# Supplementary material for: Development and external validation of a nomogram prediction model based on quantitative coronary angiography for predicting ischemic lesions: a multi-centre study
Source: Front Cardiovasc Med. 2025 Jun 20;12:1550550. doi: 10.3389/fcvm.2025.1550550 (PMC12226509; doi:10.3389/fcvm.2025.1550550)
Supplement: Supplementary file 1 [file Table1.docx]

Supplementary Material

# Supplementary Figures and Tables

## Supplementary Figures


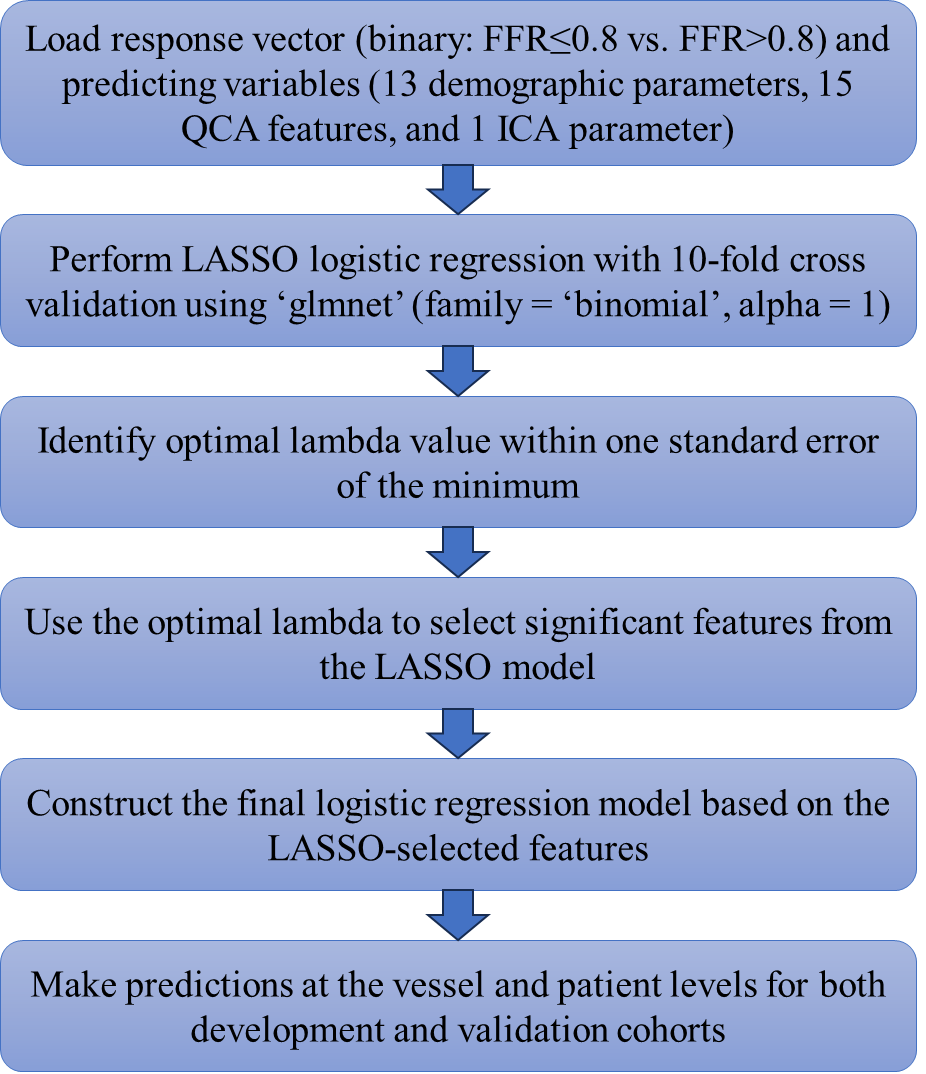


**Supplementary Figure 1.** Flowchart outlining the LASSO model training, validation, and performance evaluation. FFR, fractional flow reserve; QCA, quantitative coronary angiography; ICA, invasive coronary angiography.


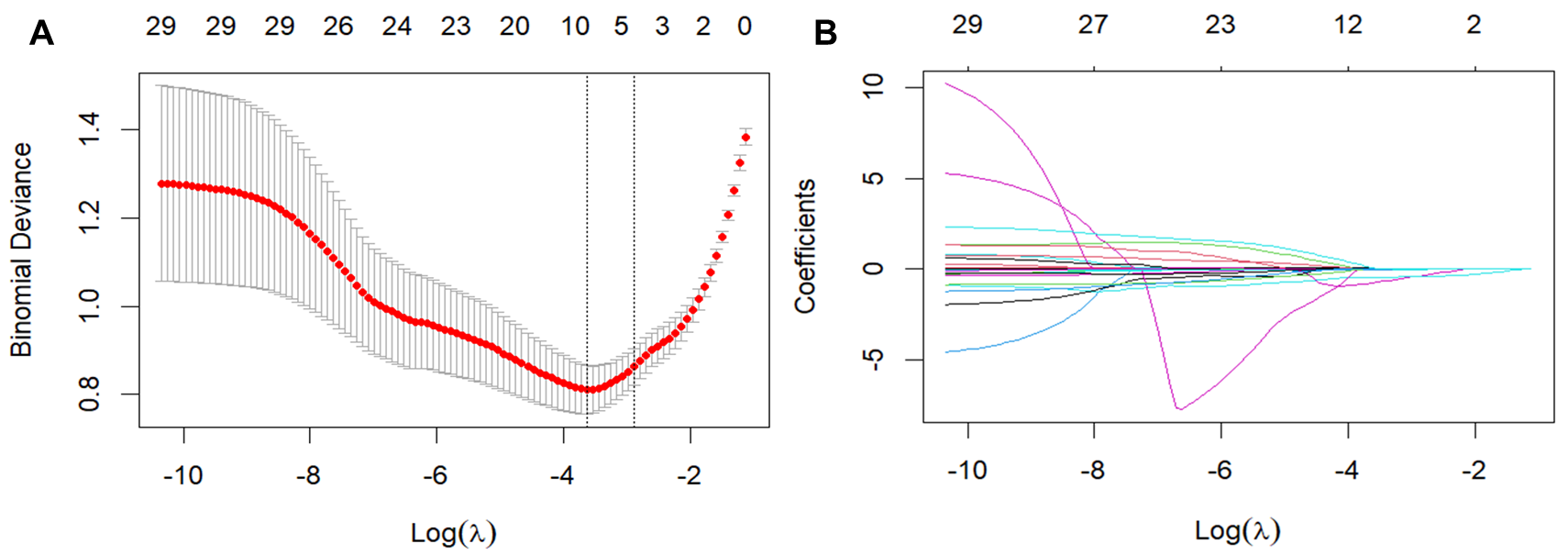


**Supplementary Figure 2**. Generation of the optimal penalization coefficient lambda. **(A)** Ten-time cross-validation for tuning parameter selection in the LASSO model. **(B)** LASSO coefficient solution path for the 5 features.


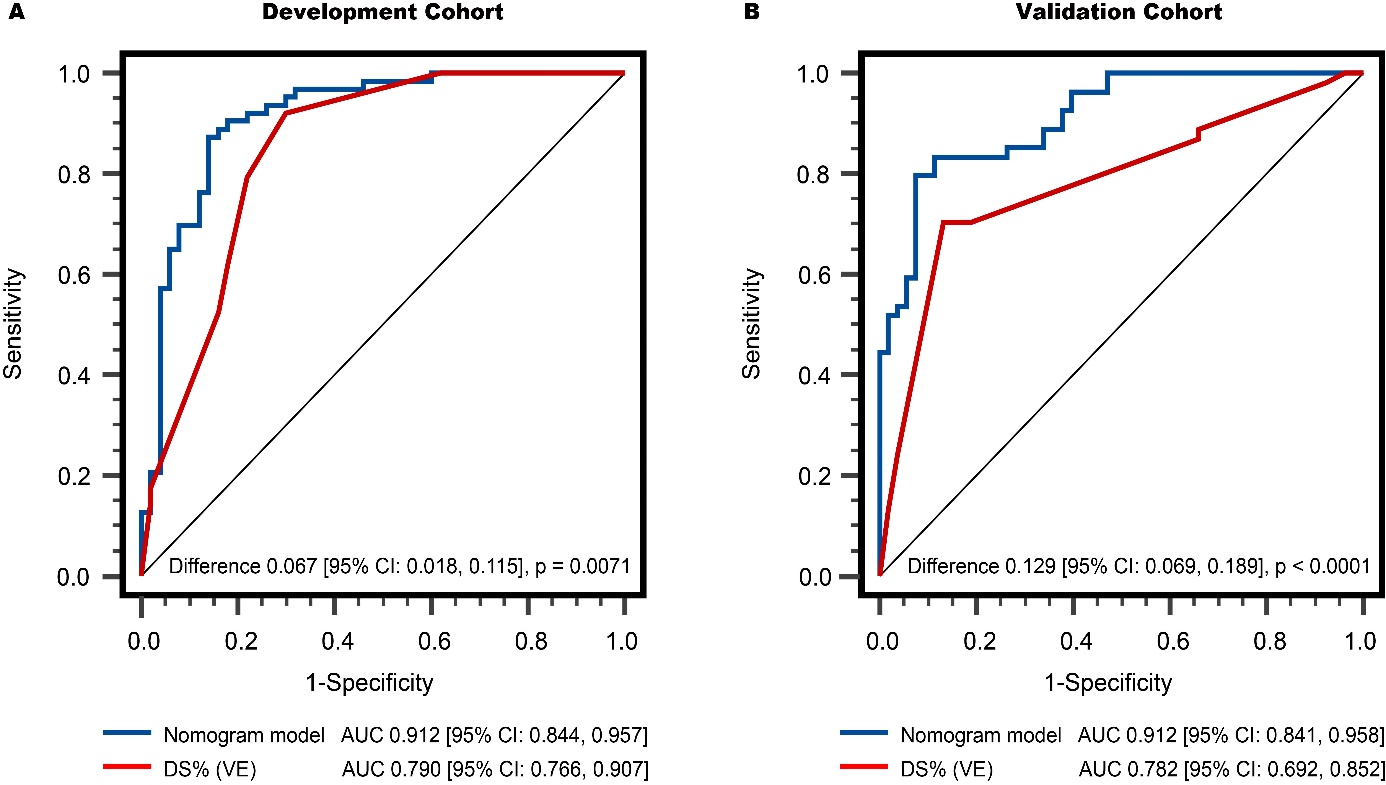


**Supplementary Figure 3.** Comparison of patient-level diagnostic performance in discriminating functionally significant stenosis: **(A)** development cohort, and (**B)** validation cohort. The AUCs of the nomogram model were both significantly higher than that of DS% (VE). AUC, areas under the receiver operator characteristics curve; DS%, percent diameter stenosis; QCA, quantitative coronary angiography; VE, visual estimation.


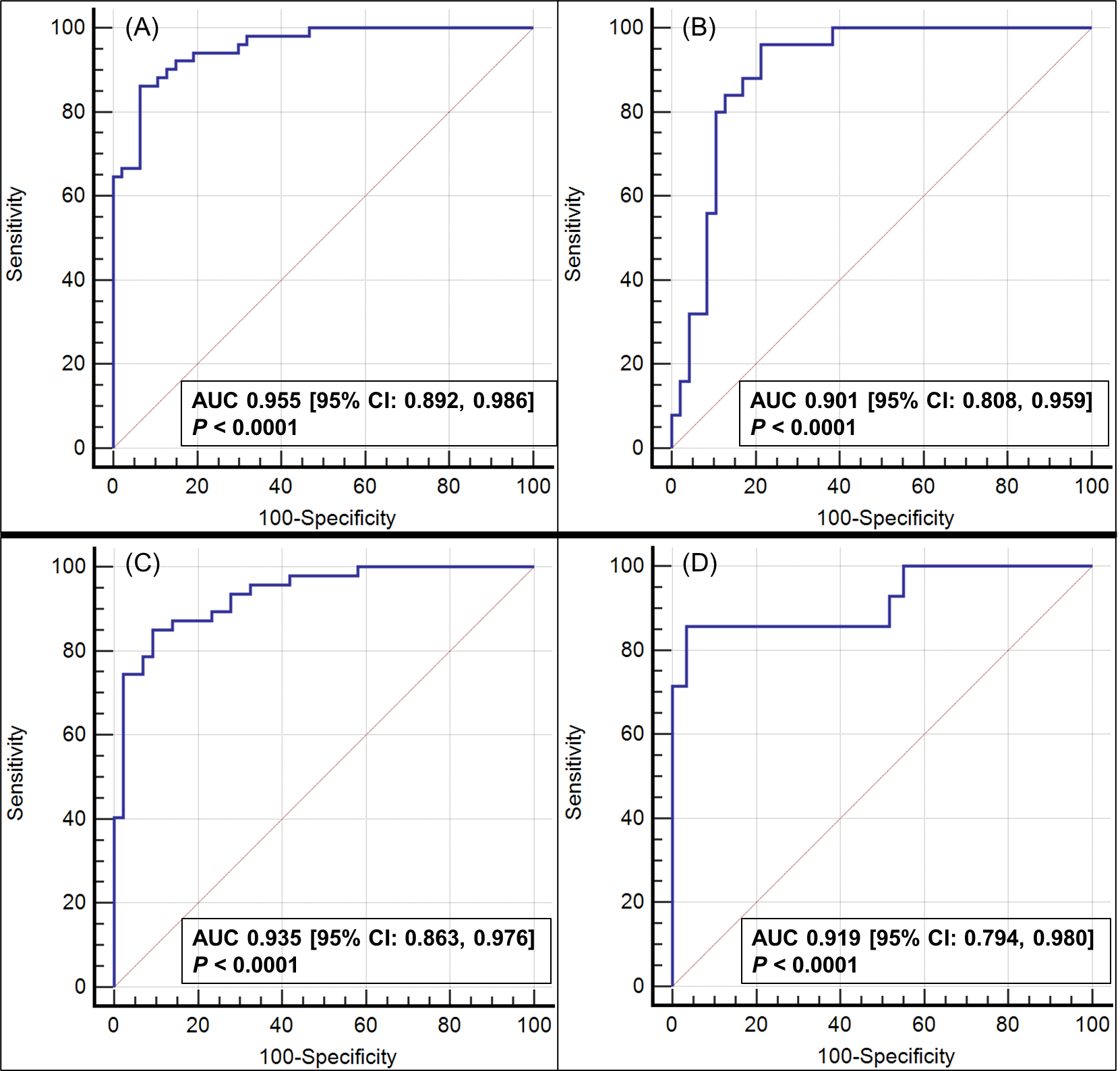


**Supplementary Figure 4.** Receiver operating characteristic (ROC) curves for LAD and non-LAD vessels. Top row: development cohort – **(A)** LAD and **(B)** non-LAD. Bottom row: validation cohort – **(C)** LAD and **(D)** non-LAD. The model shows comparable discriminatory performance across vessel types in both cohorts. LAD, left anterior descending.


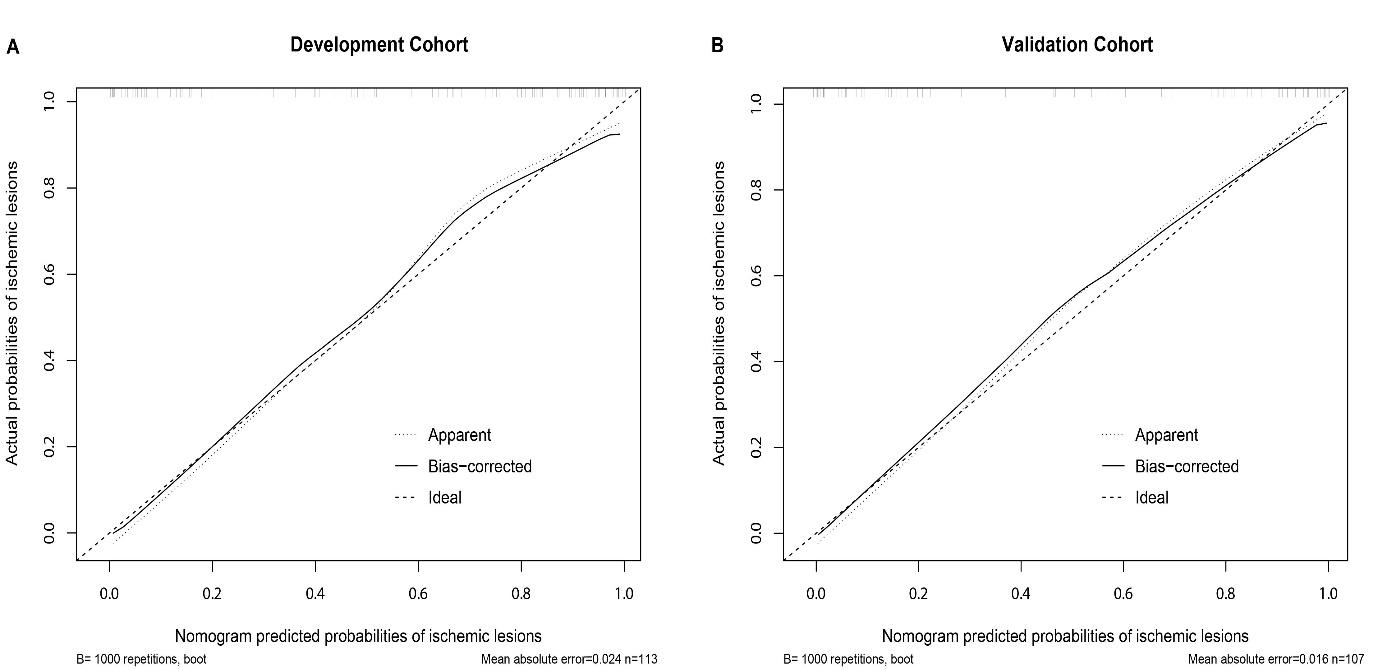


**Supplementary Figure 5.** The calibration curve of the nomogram model in estimating the risk of ischemic lesions in the **(A)** development cohort and **(B)** validation cohort.


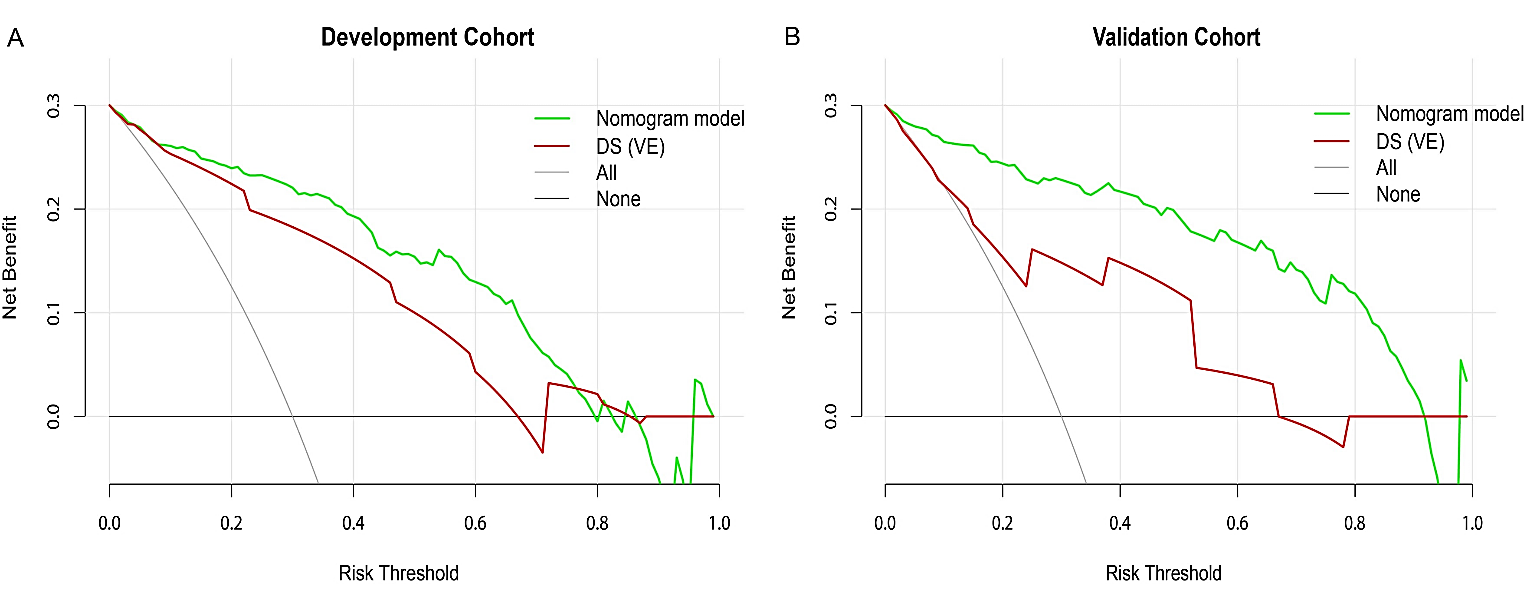


**Supplementary Figure 6.** Decision curves of the nomogram model. The x-axis measures the threshold probability. The y-axis measures the net benefit. **(A)** and **(B)** depict the decision curves predicting the presence of ischemic lesions (FFR≤0.80) generalization probabilities in the development and validation cohorts. DS (VE), diameter stenosis by visual estimation.

## Supplementary Tables

**Supplementary Table 1.** Lesion characteristics by quantitative coronary angiography (QCA) and their definitions/descriptions.

| **Parameter** | **Definition / Description** |
| --- | --- |
| **Reference lumen diameter (mm)** | Estimated diameter of the vessel at the lesion site, assuming no atherosclerotic plaque. |
| **Reference lumen area (mm^2^)** | Estimated cross-sectional area at the lesion site, assuming a disease-free vessel. |
| **Minimal lumen diameter (mm)** | Smallest diameter measured at the point of maximum stenosis. |
| **Minimal lumen area (mm^2^)** | Smallest cross-sectional area at the point of maximum stenosis. |
| **Lesion length (mm)** | Distance between the proximal and distal ends of the lesion, measured from healthy-to-healthy segments. |
| **Inflow angle (°)** | Angle at which the vessel approaches the lesion, relative to the vessel axis. |
| **Outflow angle (°)** | Angle at which the vessel exits the lesion, relative to the vessel axis. |
| **Area stenosis (%)** | Calculated as: [1 – (minimal lumen area/reference lumen area)] × 100%. |
| **Plaque symmetry** | Ratio of plaque areas on either side of the lesion plane, ranging from 0 (completely asymmetric) to 1 (completely symmetric). |
| **Plaque area (mm^2^)** | Area occupied by atherosclerotic plaque within the lesion length. |
| **Turbulent resistance** | Resistance to blood flow due to turbulence across the stenosis. See Supplementary Material (Section 2) for details. |
| **Poiseuille resistance** | Laminar flow resistance across the stenosis, based on Poiseuille’s law. See Supplementary Material (Section 2) for details. |
| **Stenosis flow reserve** | Hemodynamic impact of the stenosis, based on a quadratic relationship between flow and pressure drop. See Supplementary Material (Section 2) for details. |
| **LL/MLD^4^ (mm^-3^)** | Ratio of lesion length (LL) to the fourth power of the minimal lumen diameter (MLD). |
| **Diameter stenosis by QCA (DS_QCA_, %)** | Calculated as: [1 – (minimal lumen diameter/reference lumen diameter)] × 100% |

**Supplementary Table 2**. The subset of features ultimately selected by the LASSO algorithm.

| Feature | Feature type | Feature name | LASSO coefficients | VIF |
| --- | --- | --- | --- | --- |
| 1 | ICA feature | DS_VE_ | 0.04854472 | 1.303388 |
| 2 | QCA feature | Minimal lumen diameter | -0.37164815 | 2.084459 |
| 3 | QCA feature | Lesion length | 0.01204491 | 1.094653 |
| 4 | QCA feature | Stenosis flow reserve | -0.40086118 | 2.268929 |
| 5 | Demographic feature | Weight | -0.00569883 | 1.132015 |

ICA, invasive coronary angiography; VIF, variance inflation factor; QCA, quantitative coronary angiography; DS_VE_, percent diameter stenosis by visual estimation.

**Supplementary Table 3.** Per-patient diagnostic accuracy of nomogram model, diameter stenosis (visual estimation) and diameter stenosis (quantitative coronary angiography) in the development and validation sets.

| Development  Cohort | Nomogram model risk score ≥0.5 | DS_VE_ ≥70% |
| --- | --- | --- |
|  | **Estimate, % (95% CI)** | **Estimate, % (95% CI)** |
| Accuracy | 85.8 (78.1–91.2) | 78.8 (70.3–85.4) |
| Sensitivity | 85.7 (76.0–92.9) | 79.4 (67.0–88.1) |
| Specificity | 86.0 (72.6–93.7) | 78.0 (63.7–88.0) |
| PPV | 88.5 (77.2–94.9) | 82.0 (69.6–90.2) |
| NPV | 82.7 (69.2–91.3) | 75.0 (60.8–85.5) |
| Validation  Cohort | **Nomogram model risk score ≥0.5** | **DS_VE_ ≥70%** |
|  | **Estimate, % (95% CI)** | **Estimate, % (95% CI)** |
| Accuracy | 82.2 (73.8–88.4) | 60.8 (51.3–69.5) |
| Sensitivity | 83.3 (70.2–91.6) | 87.0 (74.5–94.2) |
| Specificity | 81.1 (67.6–90.1) | 34.0 (21.9–48.4) |
| PPV | 81.8 (68.6–90.5) | 57.3 (45.9–68.0) |
| NPV | 82.7 (69.2–91.3) | 72.0 (50.4–87.1) |

DS_VE_, diameter stenosis by visual estimation; PPV, positive predictive value; NPV, negative predictive value.

**Supplementary Table 4.** Mean intra-observer and inter-observer reliability values.

| **Parameters** | **ICC^a^ (95% CI)**  **intra-observer** | **Strength of reliability** | ***P* value** | **ICC (95% CI)**  **inter-observer** | **Strength of reliability** | ***P* value** |
| --- | --- | --- | --- | --- | --- | --- |
| Diameter stenosis (QCA) | 0.87 (0.79, 0.93) | excellent | <0.001 | 0.86 (0.75, 0.92) | excellent | <0.001 |
| Area stenosis (QCA) | 0.87 (0.79, 0.93) | excellent | <0.001 | 0.86 (0.77, 0.92) | excellent | <0.001 |
| Reference diameter | 0.72 (0.54, 0.83) | good | <0.001 | 0.73 (0.56, 0.84) | good | <0.001 |
| Reference area | 0.77 (0.62, 0.86) | excellent | <0.001 | 0.72 (0.55, 0.83) | good | <0.001 |
| **Minimal lumen diameter** | 0.94 (0.89, 0.96) | excellent | <0.001 | 0.90 (0.79, 0.95) | excellent | <0.001 |
| Minimal lumen area | 0.94 (0.89, 0.96) | excellent | <0.001 | 0.91 (0.83, 0.95) | excellent | <0.001 |
| Proximal length | 0.45 (0.18, 0.65) | moderate | <0.001 | 0.50 (0.15, 0.71) | moderate | <0.001 |
| **Lesion length** | 0.86 (0.75, 0.92) | excellent | <0.001 | 0.76 (0.71, 0.82) | excellent | <0.001 |
| Distal length | 0.49 (0.25, 0.68) | moderate | <0.001 | 0.23 (-0.03, 0.46) | poor | 0.029 |
| Inflow angle | 0.65 (0.43, 0.79) | good | <0.001 | 0.50 (0.24, 0.69) | moderate | <0.001 |
| Outflow angel | 0.72 (0.55, 0.83) | good | <0.001 | 0.41 (0.15, 0.62) | moderate | 0.002 |
| Proximal mean diameter | 0.68 (0.50, 0.81) | good | <0.001 | 0.63 (0.43, 0.78) | good | <0.001 |
| Lesion mean diameter | 0.82 (0.71, 0.90) | excellent | <0.001 | 0.71 (0.52, 0.83) | good | <0.001 |
| Distal mean diameter | 0.85 (0.75, 0.91) | excellent | <0.001 | 0.66 (0.46, 0.79) | good | <0.001 |
| Proximal area | 0.31 (0.05, 0.54) | poor | <0.01 | 0.46 (0.18, 0.67) | moderate | <0.001 |
| Lesion area | 0.86 (0.75, 0.92) | excellent | <0.001 | 0.73 (0.56, 0.84) | good | <0.001 |
| Distal area | 0.48 (0.23, 0.67) | moderate | <0.001 | 0.24 (-0.03, 0.47) | poor | 0.036 |
| Plaque symmetry | 0.42 (0.16, 0.63) | moderate | <0.01 | 0.47 (0.21, 0.66) | moderate | <0.001 |
| Plaque area | 0.83 (0.72, 0.90) | excellent | <0.001 | 0.78 (0.64, 0.87) | excellent | <0.001 |
| Turbulent resistance | 0.86 (0.76, 0.92) | excellent | <0.001 | 0.74 (0.57, 0.85) | good | <0.001 |
| Poiseuille resistance | 0.82 (0.70, 0.89) | excellent | <0.001 | 0.66 (0.45, 0.80) | good | <0.001 |
| **Stenotic flow reserve** | 0.92 (0.87, 0.96) | excellent | <0.001 | 0.84 (0.72, 0.91) | excellent | <0.001 |
| LL/MLD^4^ | 0.94 (0.89, 0.96) | excellent | <0.001 | 0.64 (0.43, 0.79) | good | <0.001 |

LL, lesion length; MLD, minimum lumen diameter; LL/MLD^4^, the ratio of lesion length to the fourth power of minimum lumen diameter; ICC, intraclass correlation coefficients; CI, confidence interval.

^a^According to Landis and Koch's reports, an ICC values was categorized as excellent agreement (ICC ≥ 0.75), good agreement (0.6 ≤ ICC < 0.75), moderate (0.4 ≤ ICC < 0.6), and poor agreement (ICC value < 0.40).

# **Derivation of SFR, PR, and TR**

The pressure drop $(\Delta P)$ across a stenotic lesion can be described by a fluid dynamics equation that relates it to flow volume $(Q)$ as follows [1]:

$\Delta P=\frac{8\pi\mu L}{As}\left( \frac{1}{As} \right)Q+\frac{\rho}{2}\left( \frac{1}{As}-\frac{1}{A\eta} \right)^{2}Q^{2}=fQ+sQ^{2}$ (1)

where $\mu$ is the blood viscosity, $L$ is the lesion length, $A\eta$ is the reference lumen area, $As$ is the minimal lumen area at the site of maximal stenosis, and $\rho$ is the blood density. The coefficients $f$ and $s$ represent pressure losses due to viscous (laminar) flow and flow separation (turbulent effects), respectively.

**Stenotic flow reserve (SFR)** is defined as the ratio between maximal and resting flow $(Q_{m}/Q_{n.r})$ at a fixed aortic pressure in the presence of a stenosis. This is found at the point where the curve representing the stenosis-induced pressure drop intersects with the line representing the pressure drop across the myocardial bed:

- The quadratic function for the pressure drop across a stenosis (black curve in Supplementary Figure 7) is given by:

$P_{c}=P_{a}-fQ_{n.r}\left( \frac{Q}{Q_{n.r}} \right)-s({Q_{n.r})}^{2}\left( \frac{Q}{Q_{n.r}} \right)^{2}$ (2)

- where $P_{a}$ is the aortic pressure (assumed to be 100 mmHg), $Q_{n.r}$ is the resting flow velocity (assumed to be 15 cm/s); and $f$, $s$ are derived from Equation (1).
- The linear function for the pressure drop across the distal myocardial circulation during hyperemia (blue curve in Supplementary Figure 6) is:

$P_{c}=\frac{P_{a}-P_{v}}{{SFR}_{n}}\cdot\frac{R}{R_{m}}\cdot\frac{Q}{Q_{n.r}}+P_{v}$ (3)

where $P_{v}$ (equal to $P_{b}$) is the venous pressure or backpressure, assumed to be 10 mmHg; ${SFR}_{n}$ is the normal SFR in the absence of a stenosis, set at 5.0; and $R$ and $R_{m}$ are the coronary resistances with and without stenosis, respectively. Since the SFR is determined at the point of intersection between Equations (2) and (3), maximal vasodilation is assumed, making $R/{R_{m}=1}$.

**Poiseuille resistance (PR)** is computed using the lesion length, cross-sectional area of the stenosis, and an assumed constant for blood viscosity. **Turbulent resistance (TR)**, on the other hand, is estimated based on the difference between the stenotic and reference lumen areas, incorporating assumed values for blood velocity and density [2].

1. Kirkeeide RL, Gould KL, Parsel L. Assessment of coronary stenoses by myocardial perfusion imaging during pharmacologic coronary vasodilation. VII. Validation of coronary flow reserve as a single integrated functional measure of stenosis severity reflecting all its geometric dimensions. *J Am Coll Cardiol*. (1986) 7:103-13. doi: 10.1016/s0735-1097(86)80266-2
2. Potter EL, Machado C, Malaiapan Y, Narayan O, Ko BS, Psaltis PJ, et al. Stenotic flow reserve derived from quantitative coronary angiography has modest but incremental value in predicting functionally significant coronary stenosis as evaluated by fractional flow reserve. *Cardiovasc Diagn Ther*. (2017) 7:52-9. doi: 10.21037/cdt.2016.12.01


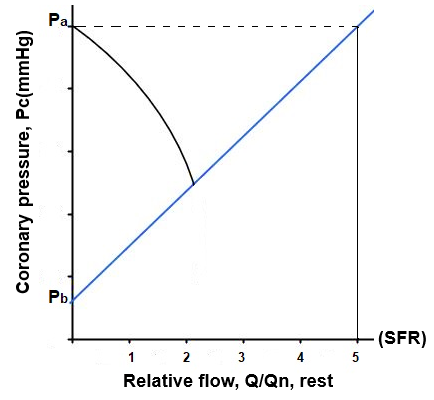


**Supplementary Figure 7.** A quadratic function of flow describing pressure drop across a stenosis and a linear function of flow describing the pressure difference across the downstream myocardial bed during hyperemia.
